# Green Extraction of Phenolic Compounds from *Aronia melanocarpa* Using Deep Eutectic Solvents and Antioxidant Activity Investigation

**DOI:** 10.3390/antiox14010031

**Published:** 2024-12-29

**Authors:** Maja Molnar, Martina Jakovljević Kovač, Lidija Jakobek, Lovro Mihajlović, Valentina Pavić

**Affiliations:** 1 Faculty of Food Technology Osijek, Josip Juraj Strossmayer University of Osijek, F. Kuhača 18, 31000 Osijek, Croatia; maja.molnar@ptfos.hr (M.M.); mjakovljevic@ptfos.hr (M.J.K.); ljakobek@ptfos.hr (L.J.); 2 Department of Biology, Josip Juraj Strossmayer University of Osijek, Cara Hadrijana 8/A, 31000 Osijek, Croatia; lovro.mihajlovic@biologija.unios.hr

**Keywords:** chokeberry, phenolic compounds, antioxidant activity, deep eutectic solvents, extraction

## Abstract

This study explores the green extraction of phenolic antioxidants from *Aronia melanocarpa* fruit using choline-chloride-based deep eutectic solvents (DESs) as an eco-friendly alternative to conventional solvents. Sixteen DESs, prepared by combining choline chloride with various hydrogen bond donors, were characterized for their physical properties, including viscosity, polarity, and pH, and applied to extract phenolics from *Aronia melanocarpa*. High-performance liquid chromatography (HPLC) quantified key phenolic compounds, including neochlorogenic and chlorogenic acid, quercetin derivatives, and cyanidin derivatives, as well as total phenolic acids, flavanols, and anthocyanins. The results revealed that DES composition and physical properties significantly influenced extraction efficiency and antioxidant activity. Additionally, the intrinsic antioxidant activity of DESs contributed substantially to the overall activity of the extracts, particularly in DESs containing organic acids or thiourea. Choline chloride/tartaric acid DES demonstrated the highest total phenolic content, attributed to its high viscosity and strongly acidic pH, while choline chloride/thiourea DES, with low viscosity and slightly acidic pH, exhibited the greatest antioxidant activity. This study highlights how tailoring DES formulations can optimize the extraction of target compounds while accounting for the solvent’s intrinsic properties. The findings support the potential application of DESs as environmentally friendly solvents in the food, pharmaceutical, and cosmetic industries.

## 1. Introduction

The rising demand for natural antioxidants in various industries, such as food, cosmetics, and pharmaceuticals, has highlighted the need for sustainable extraction methods for bioactive compounds [1,2]. Plant-based sources, rich in polyphenols, flavonoids, tannins, and vitamins, are recognized for their potent antioxidant properties, which help combat oxidative stress and extend product shelf life [3,4]. Efforts to maximize yield while minimizing environmental impact have aligned with global movements toward green chemistry and sustainable resource management [5].

One prominent source of natural antioxidants is *Aronia melanocarpa* L., commonly known as black chokeberry. Renowned for its health-promoting properties, chokeberry is an exceptional source of dietary fiber, vitamins, phenolic compounds, and minerals [6,7]. Polyphenolic compounds are the most prominent bioactive components of this fruit, which constitute up to 3.5% of its composition [8]. These compounds not only impart the fruit’s deep purple color but also contribute to its potent antioxidant activity [9], cardiovascular benefits [10], anti-inflammatory effects [11], and potential neuroprotective roles [12]. Moreover, chokeberry extracts have shown promise as natural food colorants and antibacterial agents, broadening their applications [7,13,14].

Despite its rich antioxidant profile, the astringent taste of chokeberry limits its fresh consumption, with products like juices, jams, and teas being more common [6,15].

Anthocyanins, a subgroup of flavonoids, play a vital role in protecting the cardiovascular system [16,17] by improving endothelial function [18], reducing blood pressure [19], and lowering cholesterol levels [20]. Furthermore, flavonoids and other phenolic acids found in *A. melanocarpa* have been shown to have anti-inflammatory and neuroprotective effects, making them promising agents in managing conditions like Alzheimer’s disease [21,22]. *A. melanocarpa* is especially rich in anthocyanins, which exhibit various biological activities [7,23], as shown in Table 1. Notably cyanidin-3-galactoside, the most abundant anthocyanidin [7], is accompanied by cyanidin-3-glucoside, cyanidin-3-arabinoside, and cyanidin-3-xyloside [14,24]. Additionally, chlorogenic and neochlorogenic acids are the predominant phenolic acids in chokeberry, contributing to its bioactivity [15]. Studies have demonstrated that regular consumption of *A. melanocarpa* extracts can reduce markers of oxidative damage and improve antioxidant status in the body [21,25,26]. This powerful phenolic profile not only provides a natural defense against oxidative stress but also supports metabolic and immune health [21,23,27,28], making *A. melanocarpa* a valuable dietary component in preventive health strategies.

Chokeberry also exhibits potent antibacterial effects [29,30]. Its anthocyanins demonstrated strong antibacterial activity against *Escherichia coli* by disrupting cell wall and membrane integrity, leading to leakage of intracellular contents. Additionally, chokeberry anthocyanins bound to bacterial DNA, interfering with DNA function and cellular metabolism, ultimately resulting in cell death [14]. This multi-targeted mechanism highlights chokeberry anthocyanins’ potential as natural food preservatives.

Conventional extraction of polyphenols often relies on organic solvents like ethanol and methanol, frequently acidified to stabilize anthocyanins [7,31,32,33]. However, these methods pose environmental concerns due to solvent toxicity, disposal challenges, and potential contamination [34], underscoring the need for greener alternatives. As a response, deep eutectic solvents (DESs) have emerged as eco-friendly alternatives, offering enhanced selectivity and improved yields of bioactive compounds [35]. DESs are formed by combining hydrogen bond donors (HBDs) and hydrogen bond acceptors (HBAs) in specific molar ratios, resulting in stable intermolecular interactions, primarily through hydrogen bonding [36,37,38]. These interactions substantially lower the mixture’s melting point, allowing it to remain in a liquid state at room temperature [36,39,40]. DESs offer numerous advantages, including easy preparation, short extraction time, nonflammability, low vapor pressure, and tunable polarity and viscosity depending on the chosen components [41]; therefore, they are used across various fields, particularly in the extraction of diverse bioactive compounds [42]. Compared to conventional organic solvents, DESs provide enhanced selectivity and improved extraction yields [43], often attributed to their ability to disrupt lignocellulosic structures in plant cell walls, facilitating mass transfer [44]. However, one of the most significant benefits of using deep eutectic solvents (DESs) in extraction processes is the extensive range of HBA and HBD combinations available for solvent preparation. Given that the initial components as well as their molar ratio significantly affect the DESs’ physicochemical properties [45], each DES is inherently unique. This versatility has led to DESs being described as “designer solvents”, tailored to maximize the extraction yield of desired groups of bioactive compounds [37,42]. According to numerous research, DESs have been effective in extracting a broad range of bioactive compounds, including phenolic compounds (phenolic acid and flavonoids), alkaloids, carotenoids, and proteins from various natural products and by-products, underscoring their remarkable versatility and adaptability [42,46,47].

While several studies have investigated the use of choline-chloride-based DESs for polyphenol extraction from *A. melanocarpa*, this study provides a novel contribution by addressing key aspects that remain underexplored. Some promising studies include the work by Da Silva et al. (2020), who identified a ternary Natural Deep Eutectic Solvent (NADES) formed of choline chloride/glycerol/citric acid (0.5:2:0.5) as the optimal one yielding 76% of the total anthocyanin content [48]. Similarly, Islamčević Razboršek et al. (2020) used four choline-chloride-based NADESs in combination with ultrasound to extract phenolic compounds from chokeberry. Choline chloride/d-(-)-fructose/water (2:1:1) NADES was found to be the most efficient in extraction of different phenolic compounds even compared to 80% methanol. According to authors, this could be due to strong hydrogen bonding forming between the NADES and phenolic compounds [49]. Furthermore, Lin et al. (2022) combined ultrasound-microwave-assisted extraction (UMAE) with DESs, investigating eight DESs, including two ternary formulations [50].

This study aims to evaluate choline-chloride-based DES as a sustainable and effective extraction medium for phenolic compounds from *A. melanocarpa*, offering an eco-friendly alternative to conventional solvents while optimizing the recovery of valuable antioxidants. The novelty of our study lies in its comprehensive approach, which not only evaluates the efficiency of choline-chloride-based DESs in extracting polyphenolic antioxidants but also systematically assesses the intrinsic antioxidant properties of DESs. By quantifying the contribution of DESs to the total antioxidant activity of the extracts, this study highlights their dual role as both solvents and active contributors to antioxidant potential. Additionally, this study explores the influence of DES physical properties (e.g., viscosity, polarity, and pH) on extraction efficiency and antioxidant activity, providing deeper mechanistic insights into their performance. Furthermore, our research emphasizes the scalability and sustainability of DES-based extractions, addressing limitations such as the high viscosity of certain DESs and proposing practical strategies for their optimization. This dual focus on extraction efficiency and the functional role of DESs broadens the scope of previous studies and offers valuable insights for their application in food, pharmaceutical, and cosmetic industries.

**Table 1:** Bioactive compounds in *A. melanocarpa* extracts, extraction conditions, and their biological activity.

Plant Material	Extraction Conditions	Extracted Compounds	Content	Biological Activity	Reference
pomace	Pressurized liquid ultrasound-assisted extraction, 70 °C, 180 bar, 1.5% wt. citric acid in water, min, 200 W, ultrasound bath	antocyanins: cyanidin-3-*O*-galactoside, cyanidin-3-*O*-glucoside, cyanidin-3-*O*-arabinoside, and cyanidin-3-*O*-xyloside	88%		[6]
	Ethanol	anthocyanins		prevention and treatment of type 2 *diabetes mellitus*	[23]
	0.1% HCl-acidified ethanol (55%, *v*/*v*), 1:5, 500 W ultrasonic bath, 45 °C, 90 min	anthocyanins: cyanidin 3-galactoside		anti-inflammatory activity against pulmonary disease	[7]
stems	SWE: 40 bar, 140 °C, 30 min, N_2_	TPC; TFC; rutin	48.62 mg CAE/g; 39.19 mg RE/g; 7.335 mg/g extract	cytotoxic, antibacterial, antioxidant	[15]
stem, leaves, berries	SWE 130 °C, 35 bar, 20 min, 1:20 (sample/solvent)	TPC; TFC	Leaves: 131.53 mg CAE/g, 88.64 mg RE/g; Stems: 49.96 mg CAE/g, 25.10 mg RE/g; Berries: 13.88 mg CAE/g, 10 mg RE/g	enzyme inhibiting, cytotoxic, antibacterial, antioxidant	[51]
berries	0.1 g (freeze-dried berries), 5 mL NADES, ultrasonic bath 40 kHz, 50 min, r.t.	total anthocyanin content	76%		[48]
berries	Water, shaking, 60 °C, 1 h	sugars, organic acids, polyphenols, anthocyanins	total polyphenols 11,237.4 mg/L; anthocyanins 2125.1 mg/L	neuroprotective effect in aged rats	[12]
berries	100 g, 300 mL H_2_O, Thermostatic shaker water bath, 1 h, 60 °C	TPC, anthocyanins	53.5 mg/g extract; 14.8 mg/g extract	antioxidant, antimicrobial	[52]
berry	UAE, 75% ethanol	anthocyanins		antibacterial on *E. coli*	[14]
dried berries	distilled water 0.25:10, 10 min; 90 °C			antioxidant	[53]
berries	orbital shaker (170 rpm), 50% ethanol, 60 min solid/solvent 1:20	phenolic compounds	Cyanidin-3-galactoside compound 0.34 mg/g, cyanidin-3-arabinoside 0.16 mg/g, cyanidin-3-glucoside 0.06 mg/g, quercetin-3-glucoside 0.15 mg/g, quercetin-3-galactoside 0.31 mg/g, quercetin-3-rutinoside 0.28 mg/g, chlorogenic acid 3.53 mg/g, total proanthocyanidins 36.5 mg CE/g	immunomodulatory effect	[54]
berries	5 g, 20 mL MeOH acidified with HCl (0.1%, *v*/*v*), 2 min, 16 h r.t., dark; 5 g, 10 mL hot methanol, 2 min	total anthocyanins; total polyphenols	4341.06 mg/kg 10,637 mg/kg	antioxidant	[31]
pulp	10 g, 500 mL 0.1% HCl-acidified ethanol solution (55%, *v*/*v*), 90 min, 45 °C, ultrasonic-assisted extraction	anthocyanins		inhibition of weight gain	[32]
peel, flesh	Methanol acidified with 0.5% trifluoroacetic acid, ultrasonic bath, solid/liquid 1:20	TPC; TAC	150 mg GAE/g dry peel, 79 mg GAE/g dry flesh; 99 mg CGalE/g dry peel, 8.5 mg CGalE/g dry flesh	antiradical	[33]
air-dried chokeberries	1 g, 5 mL NADES, ultrasonic bath, 20 min, 35 °C	TPC, TFC, TAC	36.15 GA g^−1^ DW, 4.71 mg RUT g^−1^ DW, 1.01 s mg Cya-3-Glu g^−1^ DW		[49]

SWE: subcritical water extracts; TPC: total phenolic content; TFC: total flavonoid content; RE: rutin equivalent; GAE: gallic acid equivalents; CGalE: cyanidin 3-*O*-galactoside equivalents; CAE: chlorogenic acid equivalents.

## 2. Materials and Methods

### 2.1. Chemicals

Phenolic standards were purchased from Sigma-Aldrich, St. Louis, MO, USA (quercetin-3-rutinoside hydrate, quercetin-3-glucoside, neochlorogenic acid, and chlorogenic acid) and from Extrasynthese, Genay, France (cyanidin-3-galactoside chloride, cyanidin-3-glucoside chloride, and quercetin-3-galactoside).

### 2.2. Preparation of DESs

The deep eutectic solvents (DESs) were prepared following the method described in our previous work [55] by combining a hydrogen bond donor (HBD) and a hydrogen bond acceptor (HBA) in a predetermined molar ratio, as outlined in Table 2. The mixture was heated to 80 °C until a clear, homogeneous liquid formed. All the DESs were diluted with water prior to the extraction process (*v*/*v* 80/20).

### 2.3. Physical Properties of DESs

#### 2.3.1. Viscosity Measurement

The viscosity of all DES–water mixtures (80:20 (*v*/*v*)) was determined using a V-COMPACT L viscometer (Fungilab S.A., Sant Feliu de Llobregat, Barcelona, Spain) over the temperature range of 30–70 °C. The solvents were gradually heated using a flow bath during the measurements. Each measurement protocol lasted 30 min, with the rotation speed of the piston (TL7) varying between 20 and 250 rpm throughout the period. Prior to each measurement, the solvents were heated to the target temperature, which was then maintained throughout the measurement period. Viscosity measurements were taken every 5 s, and the resulting data were used to generate viscosity curves for each solvent. A volume of 20 mL of each solvent was used per measurement, and each measurement was performed in triplicate to ensure reproducibility.

#### 2.3.2. Polarity Determination

The polarity of all DES–water mixtures (80:20 (*v*/*v*)) was assessed using Nile Red (9-diethylamino-5H-benzo[a]phenoxazin-5-one) dye, following the procedure outlined by Craveiro et al. (2016) [56]. An ethanolic solution of Nile Red (1 mg/mL) was prepared, and 5 µL of this solution was added to a cuvette containing 1 mL of the eutectic solvent. After thorough mixing, the UV spectra were recorded immediately using a UV-1280 UV-VIS Spectrophotometer (Shimadzu, Kyoto, Japan). The maximum absorption wavelength (*λ*_max_) for each sample was determined, and the molar transition energy (E_NR_) was calculated using Equation (1):

(1) $$E_{NR}=\frac{28591}{\lambda_{max}}$$

#### 2.3.3. pH Measurement

The pH of all DES–water mixtures (80:20 (*v*/*v*)) was determined using a SevenCompact Duo pH meter (Mettler-Toledo, Greifensee, Switzerland) coupled with an InLab Viscous Pro-ISM electrode. pH measurements were carried out at a constant temperature of 30 °C.

### 2.4. Extraction Procedure

A weighed portion of fresh chokeberry fruit (50 mg) was aliquoted, frozen, and later mixed with 1 mL of extraction solvent composed of the DES and ultrapure water (Millipore Simplicity 185, Darmstadt, Germany) in an 80:20 (*v*/*v*) ratio. The extraction was performed using an aluminum block (20 × 2 mL, Stuart SHT1) on a magnetic stirrer set to 1500 rpm for 60 min at 50 °C. The same extraction procedure was applied to all samples using different solvents. After extraction, the samples were centrifuged for 10 min at 6000 rpm, decanted, and stored at −18 °C until further analysis.

### 2.5. Chemical Characterization of the Extracts

HPLC system was used to characterize phenolic compounds (1260 Infinity II, with a quaternary pump, a PDA detector, a vial sampler, a Poroshell 120 EC C-18 column (4.6 × 100 mm, 2.7 µm), and a guard column Poroshell 120 EC-C18 4.6 mm) (Agilent technology, Santa Clara, CA, USA). Phenolic compounds were separated by using 0.5% H_3_PO_4_ (mobile phase A) and 100% acetonitrile (mobile phase B) and the following gradient: 5% B 0 min, 11% B 5 min, 15% B 7.5 min, 17.5% B 17.5 min, 20% B 20 min, 30% B 30 min, 70% B 32 min, 70% B 34 min, 5% B 36 min, and 5% B 38 min, with a flow of 0.5 mL/min. Phenolic acids were scanned at 320 nm, flavonols at 360 nm, and anthocyanins at 510 nm. The comparison of UV/Vis spectra and retention times of peaks in samples with those of standards were used for the identification. Quantification was conducted by using calibration curves of authentic standards. Cyanidin-3-arabinoside and cyanidin-3-xyloside were tentatively identified with the help of literature data [57] and quantified by using cyanidin-3-glucoside calibration curve.

### 2.6. Determination of Antioxidant Activity

In the present work, the antioxidant activity of *A. melanocarpa* extracts obtained in different DESs was evaluated based on DPPH, FRAP, and ABTS assays. Additionally, the antioxidant activity of pure DESs was tested under the same conditions to account for their intrinsic properties. The results for pure DESs were expressed as mmol TE per mL of DES and as DES influence on total activities (%), providing insight into their contribution to the total antioxidant capacity of the extracts.

#### 2.6.1. DPPH Assay

The antioxidant activity of extracts was evaluated based on their antiradical activity against 2,2-diphenyl-1-picrylhydrazyl (DPPH) using the method of Brand Williams et al. [58], adapted to 96-well microplates as described by Custódio et al. [59]. The assay mixture consisted of 200 µL of methanolic DPPH solution (120 µM) and 22 µL of the test sample. The extracts were tested in serial dilutions starting at 1.66 mg/mL, with all dilutions prepared using distilled water directly in the 96-well plate. A Trolox standard curve was prepared by serially diluting Trolox (starting at 2 mM) with distilled water. Negative, positive, and color controls were co-assayed in each plate. After 30 min of incubation at room temperature in the dark, absorbance was measured at 517 nm using a Tecan Spark Multimode Microplate Reader (Tecan Trading AG, Männedorf, Switzerland). All measurements were performed in triplicate, and DPPH scavenging activity was expressed as mmol Trolox equivalents (TE) per 100 g of fresh weight (FW).

#### 2.6.2. FRAP Assay

The ferric ion reducing antioxidant power (FRAP) assay was conducted with modifications to the method of Benzie and Strain [60]. A 10 mM solution of 2,4,6-Tripyridyl-S-triazine (TPTZ) was prepared by dissolving TPTZ in 40 mM HCl, and a 20 mM solution of iron(III) chloride hexahydrate was prepared in distilled water. The FRAP reagent was made by mixing acetate buffer (pH = 3.6), TPTZ solution, and iron(III) chloride hexahydrate solution in appropriate proportions. Serial dilutions of the samples were prepared with distilled water directly in the 96-well plate. A Trolox standard curve was prepared by serially diluting Trolox (starting at 2 mM) with distilled water. For the assay, 225 µL of FRAP reagent or acetate buffer (negative control) was added to a 96-well plate and incubated at 37 °C in the dark for 15 min. Then, 5 µL of each sample, standard, or distilled water (negative control) was added to each well containing FRAP reagent or acetic buffer. The plates were incubated on a thermoshaker at 37 °C for 30 min at 500 rpm in the dark. Absorbance was measured at 593 nm using a Tecan Spark Multimode Microplate Reader (Tecan Trading AG, Männedorf, Switzerland). All measurements were performed in triplicate, and the results were expressed as mmol TE per 100 g of FW.

#### 2.6.3. ABTS Assay

The 2,2′-azino-bis(3-ethylbenzothiazoline-6-sulfonic acid (ABTS) assay was performed following the method of Meera et al. [61], with modifications. ABTS and potassium persulfate were dissolved in distilled water to final concentrations of 7 mM and 2.45 mM, respectively. The ABTS reagent was prepared by mixing ABTS with potassium persulfate and incubating the mixture in the dark for 12 h. The reagent was then diluted with acetate buffer to achieve an absorbance of 0.74 ± 0.03 at 734 nm. Serial dilutions of the samples were prepared with distilled water directly in the 96-well plate. A Trolox standard curve was prepared by serially diluting Trolox (starting at 2 mM) with distilled water. For the assay, 200 µL of ABTS reagent was added to each well containing the sample, standard, or water (negative control) in a 96-well plate. The plates were incubated at room temperature in the dark on a thermoshaker at 500 rpm for 6 min. Absorbance was measured at 734 nm [62] using a Tecan Spark Multimode Microplate Reader (Tecan Trading AG, Männedorf, Switzerland). All measurements were performed in triplicate, and the results were expressed as mmol TE per 100 g of FW.

### 2.7. Statistical Analysis

The analyses were performed in triplicate, with results expressed as means ± standard deviation (SD). A one-way ANOVA followed by Tukey’s HSD (equal variances) and Games–Howell (unequal variances) post hoc tests was conducted to evaluate statistical differences. The normality of the distribution of numeric variables was tested by the Shapiro–Wilk test. Since data do not follow the normal distribution, the comparison of physical properties of prepared DES with the concentration of phenolic compounds and antioxidative activities of *A. melanocarpa* DES extracts was performed using the nonparametric Spearman coefficient of correlation. Data were processed using the statistical software STATISTICA 12.0 (Statsoft, Inc., Tulsa, OK, USA), with a significance level of α = 0.05 for all tests.

## 3. Results and Discussion

### 3.1. Physical Properties of Prepared DESs

The extraction potential of DESs was investigated using 16 different choline-chloride-based DESs, and the antioxidant activity of obtained extracts was determined. DESs exhibit varying physical and chemical properties depending on their components and the ratio used for their preparation, which significantly influenced the extraction efficiency observed in this research. The available data on their properties, pH, viscosity, and polarity, namely, are often limited to certain component ratio and working temperature. Therefore, hereby we determined their properties, focusing on the addition of 20% water (*v*/*v*) to each DES and a temperature of 50 °C. To reduce viscosity and enhance the diffusion rate during extraction, 20% of water was added to the DESs, as many choline-chloride-based DESs are highly viscous at room temperature [63]. Sugar, alcohol, urea, and carboxylic-acid-based DESs (Table 2) were chosen for this investigation to comprehend a broad variety of physical and chemical DES properties.

The physical properties of DESs used in this study, including viscosity, polarity, and pH, demonstrated significant variability depending on the hydrogen bond donor (HBD) (Table 3), highlighting the influence of HBD composition on their functional characteristics and extraction efficiency. Viscosity ranged widely from 10.85 ± 0.25 mPa·s for DES9 (glycerol as HBD) to 4038.00 ± 28.7 mPa·s for DES11 (malic acid as HBD), with significant differences observed among the samples (*p* < 0.001). DESs with sugar-based HBDs, such as DES4 (glucose) and DES5 (xylitol), exhibited higher viscosities (1038.90 ± 14.1 mPa·s and 207.00 ± 7.3 mPa·s, respectively). In contrast, DESs with alcohol-based HBDs, such as DES9 (glycerol) and DES7 (butane-1,4-diol), had significantly lower viscosities.

Polarity values, expressed in terms of molar transition energy (E_NR_), were relatively consistent across DESs, ranging from 44.56 for DES12 (tartaric acid as HBD) to 49.48 for DES10 (acetamide as HBD), suggesting strong hydrogen bonding and dipole interactions as defining features of these solvents. Acid-based DESs, including DES12 and DES13 (malonic acid as HBD), exhibited higher polarity, which may enhance the extraction of more polar or ionic substances, such as phenolic acids. In contrast, DES10 (acetamide), with the highest E_NR_ value, represents a lower-polarity DES and may be more effective for extracting less polar phenolic compounds. These variations in polarity demonstrate the adaptability of DESs to target specific compounds, depending on the solvent’s polar properties and the nature of the target bioactive compounds.

The pH values varied widely, from strongly acidic −0.90 for DES 14 (oxalic acid as HBD) to mildly basic 8.05 for DES 1 (urea as HBD). Acid-based DESs generally exhibited lower pH values, with DES12 (tartaric acid as HBD) and DES14 (oxalic acid as HBD) showing strongly acidic properties. This characteristic may enhance the solubility of phenolic acids and anthocyanins, as these compounds are more stable in acidic environments. DESs with alcohol or urea-based HBDs, such as DES5 and DES1, exhibited neutral to slightly basic pH values, which may limit their ability to stabilize certain bioactive compounds.

### 3.2. Chemical Profile of A. melanocarpa Extract Obtained by DES Extraction

The phenolic content in *A. melanocarpa* extracts, expressed as mg/kg fresh weight (FW), varied significantly across different deep eutectic solvents (DESs), indicating the influence of extraction medium on compound recovery (Table 4). Total phenolic content ranged from 3617.0 ± 629.9 mg/kg _FW_ with DES 9 to 7158.0 ± 22.4 mg/kg _FW_, with the highest values observed in extracts obtained using the choline chloride/tartaric acid (1:1) DES (DES12). The chromatogram of the phenolic compounds is provided in the Supplementary Materials as Figure S1. DES 12 demonstrated the highest efficiency, significantly outperforming others in extracting key phenolic compounds like neochlorogenic acid, chlorogenic acid, and anthocyanins. For instance, neochlorogenic acid content peaked at 1170.1 ± 40.0 mg/kg _FW_ in DES 12 compared to the lower values in DES 9 (598.4 ± 24.8 mg/kg _FW_). Similarly, chlorogenic acid reached its highest concentration (1428.2 ± 13.9 mg/kg _FW_) in DES 12. Islamčević Razboršek et al. (2020) obtained chlorogenic acid in the amount of 2007 mg/kg _DW_ using ChCl/lactic acid (1:2) with water (2:1 *v*/*v*), while, with methanol and ChCl/fructose DES, lower yields were obtained [49]. The anthocyanin content was also markedly higher in DES 12 (3305.7 ± 37.3 mg/kg _FW_) compared to DES 9 (1665.3 ± 484.4 mg/kg _FW_). Lin et al. (2022) applied different NADES in combination with UMAE for the extraction of anthocyanins from chokeberry. Among different NADES, ChCl/glycerol (1:6) with the addition of 30% water was the most efficient, with an extraction time of 367 s [50]. In our research, ChCl/glycerol DES was one of the least efficient solvents in extraction of phenolic compounds.

Generally, acid-based DESs showed better extraction efficacy than urea, sugar, or alcohol-based ones. For instance, neochlorogenic acid was extracted at 1170 mg/kg _FW_ using choline chloride/tartaric acid DES, which is comparable to previous studies using acidified solvents, such as Tian et al. (2017) [64] (798.8 mg/kg _FW_) and Slimestad et al. (2005) [65] (1230 mg/kg _FW_). Tian et al. (2017) utilized ethanol/water/acetic acid (70:30:1), while Slimestad et al. (2005) used methanol (0.1% HCl in methanol). The efficiency of choline chloride/tartaric acid DES in phenolic compound extraction has also been demonstrated in a prior study with propolis [66].

Chlorogenic acid was also extracted from chokeberry by Tian et al. (2017) in an amount of 1644.2 mg/kg _FW_ and Slimestad et al. (2005) in an amount of 610 mg/kg _FW_, while our research resulted in 1428 mg/kg _FW_. Other phenolic components were extracted in similarly higher or comparable yields, as shown in Table 3: quercetine-3-galactoside (171.4 mg/kg _FW_ vs. 190.9 mg/kg _FW_ [64]), quercetine-3-rutinoside (652.4 mg/kg _FW_ vs. 51.3 mg/kg _FW_ [64]), and quercetine-3-glucoside (430.2 mg/kg _FW_ vs. 127.3 mg/kg _FW_ [64]).

Anthocyanins were also effectively extracted, with cyanidine-3-galactoside reaching 1124.1 mg/kg _FW,_ compared to 3150 mg/kg _FW_ reported by Slimestad et al. (2005) [65] and 2221.1 mg/kg _FW_ by Tian et al. (2017) [64]. Cyanidine-3-glucoside was obtained at 389.2 mg/kg _FW_ (compared to 100 mg/kg _FW_ [65] and 108.7 mg/kg _FW_ [64]), cyanidine-3-arabinoside at 1346 mg/kg _FW_ vs. 1460 mg/kg _FW_ [65] and 1592.1 mg/kg _FW_ [64], and cyanidine-3-xyloside at 445.8 mg/kg _FW_ vs. 100 mg/kg _FW_ [65] and 99.2 mg/kg _FW_ [64]. These findings are consistent with prior studies [49,50], which demonstrated that specific DES compositions outperformed conventional solvents like methanol and ethanol in extracting phenolic compounds due to their ability to form stable interactions with target molecules. Given the green, sustainable nature of DESs and the short extraction time, our results offer a strong, eco-friendly alternative to conventional solvents while achieving competitive or superior extraction efficiencies for specific phenolic compounds.

The results underscore the importance of acidic media in the efficient extraction of the above-mentioned phenolics from chokeberry fruit, consistent with findings from other studies [7,32,33,64,65] that highlighted the role of DES composition in enhancing phenolic recovery. While DES12 (tartaric acid as HBD) exhibited the best extraction efficiency and is environmentally friendly due to its nontoxic components, its high viscosity at room temperature limits its broader applicability. Although adding water and increasing temperature can reduce viscosity, handling the solvent after cooling to room temperature remains challenging. For practical applications, DES15 (lactic acid as HBD) may be a more suitable alternative, as it is easier to handle despite yielding slightly lower extraction efficiencies. This research highlights the significant potential of DESs in optimizing phenolic recovery from plant materials and tailoring solvent properties for specific applications.

### 3.3. Antioxidant Activity of A. melanocarpa Extract Obtained by DES Extraction

The antioxidant activity of *A. melanocarpa* extracts prepared using different DESs was evaluated using DPPH, ABTS, and FRAP assays, with results expressed as mmol _TE_/100 g _FW_ (Table 5).

The extracts demonstrated significant variability in antioxidant capacity depending on the hydrogen bond donor (HBD) used in the DES formulation, emphasizing the critical role of solvent composition in bioactive compound extraction. The extract obtained with DES3 (choline chloride/thiourea) exhibited the highest antioxidant activity across all assays. It achieved a DPPH radical scavenging activity of 86.93 ± 0.53 mmol _TE_/100 g _FW_, a FRAP reducing power of 116.19 ± 11.00 mmol _TE_/100 g _FW_, and an ABTS radical scavenging activity of 204.62 ± 4.75 mmol _TE_/100 g _FW_, significantly outperforming all other DESs. Extracts obtained with acid-based DESs, such as DES11 (malic acid) and DES12 (tartaric acid), showed moderate antioxidant activity. DES11 exhibited a DPPH activity of 21.10 ± 0.35 mmol _TE_/100 g _FW_, a FRAP activity of 23.15 ± 0.46 mmol _TE_/100 g _FW_, and an ABTS activity of 17.49 ± 0.29 mmol _TE_/100 g _FW_, while DES12 showed the highest ABTS activity among acid-based DESs at 24.34 ± 0.13 mmol _TE_/100 g _FW_.

In contrast, extracts obtained with DESs with sugar or alcohol-based HBDs (e.g., xylitol and ethane-1,2-diol) displayed comparatively lower antioxidant activities, with DPPH values ranging from 8.21 ± 0.44 to 12.98 ± 0.27 mmol _TE_/100 g _FW_, FRAP values from 6.46 ± 0.38 to 16.89 ± 0.46 mmol _TE_/100 g _FW_, and ABTS values from 11.23 ± 1.47 to 15.37 ± 0.11 mmol _TE_/100 g _FW_. These results highlight the exceptional antioxidant extraction efficiency of choline chloride/thiourea DES (DES3), demonstrating its potential for applications requiring high antioxidant activity.

The antioxidant activity of pure DESs was also evaluated using DPPH, ABTS, and FRAP assays, with results expressed as mmol _TE_/ mL _DES_ and as DES influence on antioxidant assays (%) (Table 6).

In addition to the variability in antioxidant activity due to phenolic extraction, Table 6 reveals the intrinsic antioxidant activity of the DESs themselves. For example, DES3, which contains thiourea, contributed 59.56% of the total DPPH activity, 31.65% of the ABTS activity, and 15.03% of the FRAP activity. Other DESs, such as DES1 and DES5, showed minimal contributions to the total antioxidant activity, with DES contributions generally below 10%. This demonstrates that DES composition not only influences the extraction efficiency of phenolic compounds but also directly impacts the observed antioxidant activity.

The strong antioxidant activity of DES3 can thus be attributed to both its superior phenolic extraction efficiency and its significant intrinsic antioxidant properties. This synergistic effect between the DES and the phenolic compounds extracted underscores the importance of selecting appropriate DESs for optimizing antioxidant potential. In contrast, DESs with lower intrinsic antioxidant activity (e.g., sugar- and alcohol-based DESs) primarily depend on the efficiency of phenolic extraction, resulting in lower overall antioxidant activities. Jurić et al. 2021 evaluated the FRAP activity of 12 choline-chloride-based DESs and different HBDs (organic acids, sugars, and alcohols), reporting FRAP values ranging from 42.69 to 1851.95 mmol ascorbic acid equivalents per gram of DES [67]. Our findings align with their observation that organic acid-based DESs generally exhibit stronger reducing power due to their HBD structure. However, our results also highlight the importance of pH and viscosity in modulating DES performance, where lower viscosity and slightly acidic pH further enhanced antioxidant extraction.

These findings emphasize the critical role of DES composition and highlight the dual contribution of DESs as both extraction solvents and contributors to the overall antioxidant activity. This insight provides a foundation for tailoring DES formulations to specific applications where antioxidant potency is a key consideration.

The antioxidant activity of chokeberry DES extracts, assessed through DPPH, ABTS, and FRAP assays, was strongly correlated with their phenolic compounds content, as demonstrated by Spearman’s correlation coefficient analysis (Table 7). A strong positive correlation was observed between total phenolic content and antioxidant activity, particularly in the DPPH (r = 0.612, *p* < 0.001) and ABTS (r = 0.697, *p* < 0.001) assays, indicating that the phenolic composition is a primary determinant of radical scavenging ability. Among individual compounds, anthocyanins showed the strongest correlations with DPPH (r = 0.634, *p* < 0.001) and ABTS (r = 0.690, *p* < 0.001), while cyanidin-3-arabinoside also displayed strong correlations with both assays (DPPH: r = 0.631, *p* < 0.001; ABTS: r = 0.672, *p* < 0.001).

Phenolic acids and flavonols also demonstrated significant correlations with antioxidant activity, with neochlorogenic acid (r = 0.587, *p* < 0.001) and chlorogenic acid (r = 0.582, *p* < 0.001) contributing notably to radical scavenging activity in the DPPH assay. Flavonols such as quercetin-3-glucoside showed moderate contributions (r = 0.607, *p* < 0.001). However, the FRAP assay showed insignificant correlations with phenolic content (ranging from −0.081 to 0.105), suggesting it may be less sensitive to specific phenolic profiles or influenced by other nonphenolic reducing agents.

Interestingly, DES3 (thiourea as HBD) exhibited the highest antioxidant activity across all assays (DPPH: 86.93 ± 0.53 mmol _TE_/100 g _FW_; ABTS: 204.62 ± 4.75 mmol _TE_/100 g _FW_; FRAP: 116.19 ± 11.00 mmol _TE_/100 g _FW_) despite not having the highest total phenolic content, which was observed in DES12 (tartaric acid as HBD). This discrepancy may be explained by the selective extraction of specific phenolic compounds by DES3, particularly cyanidin-3-arabinoside (894.5 ± 184.3 mg/kg _FW_) and other cyanidin derivatives, which strongly correlate with antioxidant activity (Table 7). Additionally, DES3′s intrinsic antioxidant activity contributed significantly to the total antioxidant activity, accounting for 59.56% of DPPH, 31.65% of ABTS, and 15.03% of FRAP activity. In contrast, DES12, although extracting the highest phenolics content, extracted significantly higher levels of phenolic acids like neochlorogenic acid (1170.1 ± 40.0 mg/kg _FW_) and chlorogenic acid or flavonols with moderate radical scavenging potential, resulting in comparatively lower antioxidant activity and moderate correlation with antioxidant activity in the DPPH and ABTS assays.

These results align with previous studies highlighting the critical role of specific phenolic compounds in chokeberry’s antioxidant activity. Research by Denev et al. (2019) [52] demonstrated that approximately 40% of chokeberry’s antioxidant activity is attributable to procyanidins, while anthocyanins, hydroxycinnamic acids, and epicatechin contribute 24%, 18%, and 11%, respectively. Furthermore, Oszmiański and Wojdylo (2005) [68] reported higher antioxidant activities in fresh chokeberry fruit compared to juice or pomace, emphasizing the impact of processing and compound composition on antioxidant capacity.

Cyanidin-3-*O*-arabinoside, one of the key anthocyanins in chokeberry, has been identified as the most potent radical scavenger and inhibitor of pro-oxidative enzymes, such as 15-lipoxygenase and xanthine oxidase [69]. The high antioxidant activity observed in DES3 extracts may reflect its enhanced efficiency in extracting such potent bioactive anthocyanins and procyanidins, consistent with findings by Skupien and Oszmianski (2007) [70], who noted the superior antioxidant properties of procyanidins over their monomers. Additionally, Tolić et al. (2015) [71] reported the highest total antioxidant capacity in dried chokeberries, with mean values reaching 187.41 mmol _TE_/100 g _DM_, providing a comparative baseline for the present findings.

The strong correlations between phenolic content and DPPH/ABTS activities reinforce the efficiency of DES3 in selectively extracting bioactive compounds, particularly anthocyanins, that maximize antioxidant potential. These findings underscore the importance of selecting extraction media tailored to the desired functional outcomes, as DES3 demonstrates exceptional potential for maximizing antioxidant activity, while DES12 may be more suitable for applications requiring broader phenolic recovery.

The observed variations in the physical properties of DESs, including viscosity, polarity, and pH, significantly influenced their performance in phenolic extraction and antioxidant activity (Table 5). DES3 (thiourea as HBD), with low viscosity (50.70 ± 2.8 mPa·s), moderately high polarity (E_NR_ = 48.24), and slightly acidic pH (5.74 ± 0.0), probably facilitated efficient mass transfer and stabilization of phenolics, contributing to its high antioxidant activity despite moderate phenolics content. In contrast, DES12 (tartaric acid as HBD), with high viscosity (1548.50 ± 9.2 mPa·s), higher polarity (E_NR_ = 44.56), and strongly acidic pH (−0.19), excelled in broad-spectrum phenolic recovery, particularly for anthocyanins and phenolic acids, suggesting that increased molecular interactions in higher-viscosity DESs enhance the solubilization of specific phenolic compounds. Spearman’s correlation analysis revealed positive relationships between viscosity and phenolic content (e.g., neochlorogenic acid, r = 0.640, *p* < 0.001) and significant negative correlations between pH and both phenolic content (e.g., total phenolics, r = −0.692, *p* < 0.001; phenolic acids, r = −0.721, *p* < 0.001) and antioxidant activity (DPPH: r = −0.616, *p* < 0.001; ABTS: r = −0.657, *p* < 0.001), indicating that acidic DESs stabilize or enhance the reactivity of antioxidant compounds. Polarity also influenced phenolic recovery, with higher-polarity DESs (lower ENR) favoring the extraction of polar compounds like flavonols and anthocyanins. These findings emphasize the importance of optimizing polarity to align with the chemical nature of the target phenolic compounds for enhanced extraction efficiency. However, polarity showed insignificant correlations with antioxidant activity, indicating its limited role under these conditions. Interestingly, these trends were less evident in the FRAP assay, where no significant correlations were observed between physical properties and antioxidant activity, likely due to differing reaction mechanisms.

These findings underscore the importance of optimizing DES properties to align with the chemical nature of target compounds, with lower-viscosity, slightly acidic DESs like DES3 being ideal for rapid extraction of reactive antioxidants, while highly viscous, strongly acidic DESs like DES12 may be better suited for stabilizing and recovering a broader spectrum of phenolics. The relationship between polarity and phenolic recovery further highlights the adaptability of DESs for diverse applications. By optimizing viscosity, polarity, and pH, DESs can be designed to maximize the recovery and functionality of bioactive compounds, offering a versatile approach for natural product extraction.

While this study provides valuable insights into the use of DESs for phenolic extraction, it has several limitations that require further investigation. One limitation is the absence of procyanidin quantification, which restricts a comprehensive understanding of the phenolic profile and may lead to an underestimation of total phenolic content. Also, the isolation of desired compounds from DESs is recommended when these compounds are intended for specific purposes. However, the fact that DESs remain in the final extract can present challenges, including potential technical issues [72] or toxicity concerns [73,74], depending on the application. Due to the very low vapor pressure of DESs, their removal from the extract is particularly challenging. Based on our experience, the most effective approach to isolate desired compounds from such extracts is the use of adsorption resins, followed by desorption with a suitable solvent, tailored to the specific application. This method has yielded excellent results in our work, as detailed in our previous publication [75]. Further research should focus on evaluating the safety and compatibility of residual DESs to address potential concerns, which is critical for their use in food, pharmaceutical, and cosmetic applications.

## 4. Conclusions

This study underscores the importance of deep eutectic solvents as eco-friendly and efficient alternatives for the extraction of phenolic compounds from *Aronia melanocarpa*. The physical properties of DESs—viscosity, polarity, and pH—significantly influenced both the extraction efficiency and antioxidant activity of the extracts. Additionally, the intrinsic antioxidant activity of DESs themselves contributed to the overall antioxidant potential of the extracts, particularly in formulations containing thiourea or organic acids. The results revealed that the most effective DES for extracting antioxidant components from chokeberry fruit was choline chloride/tartaric acid. Acid-based DESs generally exhibited superior extraction capacity compared to sugar-, urea-, or alcohol-based DESs. Choline chloride/thiourea, with low viscosity, moderately high polarity, slightly acidic pH, and high intrinsic antioxidant activity, was highly effective for extracting anthocyanins and reactive antioxidants, making it suitable for rapid extraction processes. In contrast, choline chloride/tartaric acid, with high viscosity, higher polarity, strongly acidic pH, and moderate intrinsic antioxidant activity, demonstrated superior performance in broad-spectrum recovery of phenolic acids and anthocyanins, supporting applications where comprehensive phenolics recovery is desired. Viscosity positively correlated with phenolic content, while acidic DESs improved the phenolic recovery. Polarity exhibited an inverse relationship with phenolic content but had a limited influence on antioxidant activity. The intrinsic antioxidant properties of DESs, particularly those containing thiourea or organic acids, significantly contributed to the overall activity, highlighting the dual role of DESs as both solvents and antioxidant contributors. The green character of DESs makes them environmentally acceptable solvents for extracting phenolics from chokeberry fruit, aligning with sustainable practices in natural product recovery. However, their high viscosity, a key disadvantage, often necessitates the addition of water or the use of higher temperatures to improve extraction efficiency. These findings highlight the versatility of DESs in tailoring extraction processes for specific compounds, offering significant potential for scalable and stable applications in various industries. Future work should focus on optimizing DES formulations to balance viscosity, intrinsic antioxidant activity, and extraction performance, while expanding their industrial applicability.

## Figures and Tables

**Table 2:** List of prepared DESs with their corresponding molar ratios.

DES	HBA	HBD	Molar Ratio
1	ChCl	Urea	1:2
2	ChCl	*N*-methylurea	1:3
3	ChCl	Thiourea	1:2
4	ChCl	Glucose	1:1
5	ChCl	Xylitol	1:1
6	ChCl	Sorbitol	1:1
7	ChCl	Butane-1,4-diol	1:2
8	ChCl	Ethane-1,2-diol	1:2
9	ChCl	Glycerol	1:2
10	ChCl	Acetamide	1:2
11	ChCl	Malic acid	1:1
12	ChCl	Tartaric acid	1:1
13	ChCl	Malonic acid	1:1
14	ChCl	Oxalic acid	1:1
15	ChCl	Lactic acid	1:2
16	ChCl	Levulinic acid	1:2

**Table 3:** Physical properties of different DESs.

DES	Viscosity ^B^ (mPa·s)	Polarity ^B^ (E_NR_)	pH ^A^
1	102.10 ± 1.9 ^i^	48.62 ± 0.0 ^c^	8.05 ± 0.0 ^a^
2	101.80 ± 1.5 ^i^	48.96 ± 0.0 ^c^	5.99 ± 0.0 ^d^
3	50.70± 2.8 ^j^	48.24 ± 0.0 ^d^	5.74 ± 0.0 ^e^
4	1038.90 ± 14.1 ^c^	48.57 ± 0.0 ^c^	4.41 ± 0.0 ^i^
5	207.00 ± 7.3 ^g^	48.85 ± 0.0 ^c^	7.01 ± 0.0 ^b^
6	774.57 ± 6.2 ^d^	48.90 ± 0.0 ^c^	5.37 ± 0.0 ^g^
7	20.50 ± 0.0 ^k^	48.61 ± 0.0 ^c^	4.86 ± 0.0 ^h^
8	149.80 ± 6.3 ^h^	49.08 ± 0.0 ^b^	6.17 ± 0.0 ^c^
9	10.85 ± 0.25 ^l^	48.51 ± 0.0 ^c^	5.58 ± 0.0 ^f^
10	47.30 ± 2.2 ^j^	49.48 ± 0.0 ^a^	6.23 ± 0.0 ^c^
11	4038.00 ± 28.7 ^a^	48.19 ± 0.0 ^d^	0.55 ± 0.0 ^l^
12	1548.50 ± 9.2 ^b^	44.56 ± 0.0 ^e^	−0.19 ± 0.0 ^m^
13	319.50 ± 1.2 ^f^	44.67 ± 0.0 ^e^	0.60 ± 0.0 ^l^
14	616.80 ± 4.6 ^e^	44.67 ± 0.0 ^e^	−0.90 ± 0.0 ^n^
15	104.00 ± 0.7 ^i^	48.13 ± 0.0 ^d^	0.79 ± 0.0 ^k^
16	153.80 ± 2.1 ^h^	48.79 ± 0.0 ^c^	1.33 ± 0.0 ^j^

Means followed by different letters differ significantly, based on ^A^ Tukey’s HSD post hoc multiple comparison; ^B^ Games–Howell post hoc multiple comparison. The means’ difference is significant at the 0.05 level.

**Table 4:** Phenolic compounds in *A. melanocarpa* extracts obtained in different DESs expressed as mg/kg _FW_.

DES	Neochlorogenic Acid ^A^	Chlorogenic Acid ^B^	Quercetine-3-galactoside ^B^	Quercetine-3-rutinoside ^B^	Quercetine-3-glucoside ^A^	Cyanidine-3-galactoside ^B^	Cyanidine-3-glucoside ^B^	Cyanidine-3-arabinoside ^B^	Cyanidine-3-xyloside ^B^	Phenolic Acids ^A^	Flavonoles ^B^	Anthocyanins ^B^	* Total Phenolics ^B^
1	585.3 ± 24.1 ^c^	712.6 ± 11.1 ^c^	86.2 ± 0.7 ^b^	363.3 ± 19.2 ^b^	220.2 ± 5.0 ^c^	621.1 ± 63.5 ^b^	236.8 ± 14.8 ^b^	754.8 ± 77.8 ^b^	242.0 ± 16.1 ^d^	1297.9 ± 33.6 ^c^	669.7 ± 24.7 ^b^	1854.7 ± 171.7 ^b^	3822.3 ± 228.8 ^b^
2	619.0 ± 25.7 ^c^	774.7 ± 40.2 ^c^	87.3 ± 1.6 ^b^	395.7 ± 38.7 ^b^	225.6 ± 6.4 ^c^	706.7 ± 98.1 ^b^	220.2 ± 3.2 ^b^	858.1 ± 119.8 ^b^	262.9 ± 26.6 ^cd^	1393.7 ± 64.6 ^c^	708.6 ± 46.7 ^b^	2047.9 ± 247.0 ^b^	4150.1 ± 349.0 ^b^
3	648.9 ± 20.5 ^c^	775.1 ± 45.4 ^c^	87.0 ± 1.4 ^b^	381.8 ± 27.5 ^b^	224.4 ± 5.9 ^c^	747.0 ± 160.5 ^b^	233.6 ± 26.6 ^b^	894.5 ± 184.3 ^b^	263.7 ± 35.1 ^cd^	1424.0 ± 65.9 ^c^	693.3 ± 34.7 ^b^	2138.7 ± 406.5 ^b^	4256.0 ± 487.2 ^b^
4	625.4 ± 26.5 ^c^	723.4 ± 16.4 ^c^	85.2 ± 0.3 ^b^	344.2 ± 6.1 ^b^	218.1 ± 1.0 ^c^	555.4 ± 21.4 ^b^	188.3 ± 3.3 ^b^	663.8 ± 25.6 ^b^	222.7 ± 6.6 ^d^	1348.8 ± 42.8 ^c^	647.4 ± 7.1 ^b^	1630.3 ± 56.4 ^b^	3626.5 ± 97.4 ^b^
5	626.1 ± 30.6 ^c^	774.5 ± 20.4 ^c^	85.8 ± 0.5 ^b^	353.6 ± 4.3 ^b^	220.0 ± 1.3 ^c^	652.9 ± 25.0 ^b^	212.9 ± 23.0 ^b^	788.9 ± 48.1 ^b^	244.8 ± 10.2 ^d^	1400.6 ± 50.9 ^c^	659.5 ± 5.2 ^b^	1899.5 ± 101.3 ^b^	3959.6 ± 75.0 ^b^
6	642.9 ± 13.9 ^c^	763.5 ± 38.4 ^c^	85.8 ± 0.3 ^b^	352.9 ± 9.6 ^b^	218.0 ± 1.5 ^c^	623.5 ± 50.2 ^b^	200.4 ± 8.3 ^b^	745.0 ± 62.0 ^b^	242.0 ± 14.3 ^d^	1406.4 ± 52.3 ^c^	656.8 ± 11.4 ^b^	1810.8 ± 134.6 ^b^	3873.9 ± 168.3 ^b^
7	573.4 ± 14.8 ^c^	703.2 ± 23.1 ^c^	85.8 ± 0.5 ^b^	366.7 ± 2.5 ^b^	220.9 ± 2.7 ^c^	658.3 ± 30.8 ^b^	209.6 ± 9.1 ^b^	798.5 ± 42.6 ^b^	248.3 ± 8.6 ^d^	1276.6 ± 37.7 ^c^	673.4 ± 5.0 ^b^	1914.7 ± 89.7 ^b^	3864.8 ± 121.9 ^b^
8	606.4 ± 19.6 ^c^	739.5 ± 27.8 ^c^	86.0 ± 0.8 ^b^	364.5 ± 19.9 ^b^	221.6 ± 3.0 ^c^	657.5 ± 65.2 ^b^	203.0 ± 9.3 ^b^	785.3 ± 77.7 ^b^	247.4 ± 17.6 ^d^	1345.8 ± 40.8 ^c^	672.1 ± 23.7 ^b^	1893.2 ± 169.0 ^b^	3911.2 ± 227.6 ^b^
9	598.4 ± 24.8 ^c^	708.7 ± 77.7 ^c^	85.2 ± 1.8 ^b^	342.0 ± 34.7 ^b^	217.4 ± 7.4 ^c^	567.0 ± 177.2 ^b^	188.8 ± 27.6 ^b^	683.6 ± 233.0 ^b^	225.9 ± 46.9 ^d^	1307.1 ± 102.4 ^c^	644.6 ± 44.0 ^b^	1665.3 ± 484.4 ^b^	3617.0 ± 629.9 ^b^
10	587.8 ± 8.4 ^ab^	705.2 ± 17.6 ^c^	85.7 ± 0.9 ^b^	348.9 ± 17.5 ^b^	217.5 ± 3.8 ^c^	577.1 ± 85.3 ^b^	207.4 ± 16.3 ^b^	694.7 ± 92.1 ^b^	229.1 ± 15.2 ^d^	1293.0 ± 25.1 ^c^	652.1 ± 22.1 ^b^	1708.3 ± 208.2 ^b^	3653.4 ± 255.1 ^b^
11	1046.9 ± 59.1 ^a^	1210.6 ± 81.2 ^ab^	169.0 ± 1.8 ^a^	653.1 ± 33.4 ^a^	428.8 ± 7.1 ^ab^	929.7 ± 194.5 ^ab^	370.2 ± 38.9 ^a^	1170.5 ± 239.1 ^ab^	413.0 ± 50.1 ^ab^	2257.6 ± 140.0 ^ab^	1250.9 ± 41.7 ^a^	2883.4 ± 522.5 ^ab^	6391.9 ± 701.5 ^ab^
12	1170.1 ± 40.0 ^a^	1428.2 ± 13.9 ^a^	171.4 ± 1.9 ^a^	652.4 ± 12.1 ^a^	430.2 ± 1.7 ^a^	1124.1 ± 17.9 ^a^	389.2 ± 11.2 ^a^	1346.6 ± 23.0 ^a^	445.8 ± 9.8 ^a^	2598.3 ± 46.6 ^a^	1254.0 ± 14.0 ^a^	3305.7 ± 37.3 ^a^	7158.0 ± 22.4 ^a^
13	1022.6 ± 22.2 ^ab^	1179.1 ± 30.0 ^b^	167.8 ± 2.1 ^a^	608.1 ± 7.1 ^a^	422.8 ± 3.3 ^ab^	873.8 ± 12.7 ^b^	361.3 ± 6.1 ^a^	1080.6 ± 5.7 ^b^	397.9 ± 3.5 ^ab^	2201.8 ± 50.7 ^b^	1198.7 ± 5.0 ^a^	2713.7 ± 19.2 ^b^	6114.2 ± 54.5 ^b^
14	956.9 ± 32.5 ^b^	1109.3 ± 66.9 ^b^	168.6 ± 1.9 ^a^	600.6 ± 16.3 ^a^	419.5 ± 8.7 ^b^	840.3 ± 62.4 ^b^	351.1 ± 10.5 ^a^	1021.6 ± 69.8 ^b^	383.5 ± 13.4 ^ab^	2066.1 ± 99.3 ^b^	1188.7 ± 26.4 ^a^	2596.6 ± 155.1 ^b^	5851.4 ± 270.8 ^b^
15	1037.3 ± 46.4 ^ab^	1215.7 ± 62.3 ^ab^	167.8 ± 1.9 ^a^	625.5 ± 19.9 ^a^	425.6 ± 4.1 ^ab^	796.9 ± 132.2 ^b^	361.2 ± 40.4 ^a^	1020.2 ± 175.1 ^b^	394.9 ± 37.1 ^ab^	2253.4 ± 108.7 ^ab^	1218.87 ± 26.1 ^a^	2572.6 ± 384.7 ^b^	6044.4 ± 519.5 ^b^
16	1002.9 ± 41.1 ^ab^	1123.8 ± 75.6 ^b^	165.7 ± 1.3 ^a^	609.3 ± 23.4 ^a^	419.9 ± 1.8 ^b^	613.6 ± 110.9 ^b^	341.7 ± 34.8 ^ab^	777.2 ± 132.9 ^b^	339.0 ± 24.9 ^bc^	2126.7 ± 116.4 ^b^	1194.9 ± 26.4 ^a^	2071.5 ± 299.0 ^b^	5393.1 ± 437.2 ^b^

Means followed by different letters differ significantly, based on ^A^ Tukey’s HSD post hoc multiple comparison; ^B^ Games–Howell post hoc multiple comparison. The means’ difference is significant at the 0.05 level. Extraction was performed with frozen fruits, and the amount is expressed in mg/kg fresh weight (FW). * The total amount of phenolic compounds is expressed without the contribution of procyanidins.

**Table 5:** Antioxidant activity of *A. melanocarpa* extracts obtained in different DESs determined using DPPH, ABTS, and FRAP assays (expressed as mmol _TE_/100g _FW_).

DES-ArE	DPPH ^A^ (mmol _TE_/100 g _FW_)	FRAP ^B^ (mmol _TE_/100 g _FW_)	ABTS ^B^ (mmol _TE_/100 g _FW_)
DES1-ArE	8.21 ± 0.44 ^h^	6.46 ± 0.38 ^d^	11.23 ± 1.47 ^g^
DES2-ArE	9.78 ± 0.58 ^gh^	12.32 ± 0.71 ^d^	12.94 ± 1.52 ^fg^
DES3-ArE	86.93 ± 0.53 ^a^	116.19 ± 11.00 ^a^	204.62 ± 4.75 ^a^
DES4-ArE	11.54 ± 0.48 ^efg^	14.11 ± 0.89 ^d^	12.10 ± 0.16 ^g^
DES5-ArE	12.93 ± 0.46 ^de^	15.09 ± 0.68 ^d^	14.22 ± 0.39 ^efg^
DES6-ArE	10.95 ± 0.05 ^fgh^	15.41 ± 1.13 ^d^	12.89 ± 0.70 ^fg^
DES7-ArE	12.75 ± 0.11 ^de^	15.95 ± 0.39 ^d^	14.02 ± 1.41 ^fg^
DES8-ArE	12.86 ± 0.24 ^de^	13.42 ± 0.85 ^d^	13.08 ± 2.05 ^fg^
DES9-ArE	12.98 ± 0.27 ^de^	16.89 ± 0.46 ^d^	15.37 ± 0.11 ^defg^
DES10-ArE	10.20 ± 0.18 ^gh^	12.00 ± 0.17 ^d^	10.97 ± 0.18 ^g^
DES11-ArE	21.10 ± 0.35 ^b^	23.15 ± 0.46 ^c^	17.49 ± 0.29 ^cdef^
DES12-ArE	15.21 ± 0.51 ^d^	9.87 ± 0.96 ^d^	24.34 ± 0.13 ^b^
DES13-ArE	18.50 ± 0.32 ^c^	12.13 ± 0.20 ^d^	19.10 ± 2.62 ^cde^
DES14-ArE	18.97 ± 0.59 ^bc^	4.54 ± 0.07 ^d^	21.17 ± 0.75 ^bc^
DES15-ArE	15.12 ± 0.22 ^d^	27.21 ± 0.65 ^b^	19.60 ± 0.31 ^bcd^
DES16-ArE	12.50 ± 0.39 ^ef^	19.14 ± 3.22 ^cd^	15.30 ± 0.09 ^defg^

Means followed by different letters differ significantly, based on ^A^ Tukey’s HSD post hoc multiple comparison; ^B^ Games–Howell post hoc multiple comparison. The means’ difference is significant at the 0.05 level. DES represents the number from the DES table and “ArE” stands for *Aronia* extract.

**Table 6:** Antioxidant activity of pure DES determined using DPPH, ABTS, and FRAP assays expressed as mmol _TE_/mL _DES_ and as DES influence on antioxidant assays (%).

DES	DPPH (mmol _TE_/mL _DES_)	DES Influence on DPPH (%)	FRAP (mmol _TE_/mL _DES_)	DES Influence on FRAP (%)	ABTS (mmol _TE_/mL _DES_)	DES Influence on ABTS (%)
1	0.0003 ± 0.0001	8.28 ± 2.40	0.0001 ± 0.0000	1.48 ± 0.33	0.0002 ± 0.0000	4.38 ± 0.43
2	0.0006 ± 0.0001	11.58 ± 2.84	0.0002 ± 0.0000	3.36 ± 0.32	0.0002 ± 0.0000	2.83 ± 0.62
3	0.0259 ± 0.0004	59.56 ± 0.79	0.0087 ± 0.0002	15.03 ± 1.17	0.0214 ± 0.0005	31.65 ± 0.61
4	0.0008 ± 0.0000	13.74 ± 1.29	0.0002 ± 0.0000	2.54 ± 0.40	0.0001 ± 0.0000	0.96 ± 0.30
5	0.0005 ± 0.0001	8.23 ± 1.24	0.0002 ± 0.0000	2.47 ± 0.23	0.0002 ± 0.0001	2.62 ± 2.06
6	0.0005 ± 0.0001	9.60 ± 0.81	0.0002 ± 0.0000	2.45 ± 0.47	0.0001 ± 0.0000	1.02 ± 0.09
7	0.0005 ± 0.0001	8.30 ± 0.79	0.0002 ± 0.0000	2.60 ± 0.02	0.0005 ± 0.0001	6.54 ± 0.79
8	0.0003 ± 0.0001	4.98 ± 1.51	0.0001 ± 0.0000	1.08 ± 0.32	0.0003 ± 0.0000	5.46 ± 1.09
9	0.0007 ± 0.0001	10.21 ± 0.60	0.0002 ± 0.0000	2.32 ± 0.34	0.0002 ± 0.0001	2.51 ± 0.85
10	0.0006 ± 0.0001	12.48 ± 0.95	0.0002 ± 0.0000	3.75 ± 0.16	0.0000 ± 0.0001	0.17 ± 0.08
11	0.0001 ± 0.0001	0.62 ± 0.26	0.0001 ± 0.0000	1.25 ± 0.21	0.0004 ± 0.0001	4.04 ± 0.21
12	0.0001 ± 0.0001	1.38 ± 0.08	0.0015 ± 0.0000	31.09 ± 2.34	0.0002 ± 0.0001	1.51 ± 0.49
13	0.0008 ± 0.0001	8.5 ± 1.65	0.0003 ± 0.0000	5.64 ± 0.32	0.0004 ± 0.0001	4.42 ± 0.69
14	0.0010 ± 0.0001	10.26 ± 0.32	0.0005 ± 0.0000	22.56 ± 1.49	0.0004 ± 0.0001	3.87 ± 0.58
15	0.0002 ± 0.0001	2.77 ± 0.77	0.0002 ± 0.0000	1.28 ± 0.34	0.0005 ± 0.0001	5.52 ± 0.97
16	0.0005 ± 0.0001	7.44 ± 0.62	0.0001 ± 0.0000	1.33 ± 0.24	0.0002 ± 0.0001	4.18 ± 0.89

**Table 7:** Spearman’s correlation coefficient analysis between phenolic compounds in *A. melanocarpa* extracts obtained in different DESs, antioxidant activity, and physical properties of DESs.

	DPPH	FRAP	ABTS	Viscosity	Polarity	pH
Neochlorogenic acid	0.587 ***	0.105	0.680 ***	0.640 ***	−0.641 ***	−0.745 ***
Chlorogenic acid	0.582 ***	0.055	0.674 ***	0.575 ***	−0.599 ***	−0.689 ***
Quercetine-3-galactoside	0.558 ***	−0.054	0.684 ***	0.417 **	−0.616 ***	−0.664 ***
Quercetine-3-rutinoside	0.584 ***	0.053	0.669 ***	0.395 **	−0.609 ***	−0.681 ***
Quercetine-3-glucoside	0.607 ***	0.036	0.664 ***	0.407 **	−0.641 ***	−0.683 ***
Cyanidine-3-galactoside	0.618 ***	−0.081	0.666 ***	0.375 **	−0.612 ***	−0.563 ***
Cyanidine-3-glucoside	0.519 ***	−0.101	0.622 ***	0.365 *	−0.613 ***	−0.598 ***
Cyanidine-3-arabinoside	0.631 ***	−0.061	0.672 ***	0.384 **	−0.638 ***	−0.579 ***
Cyanidine-3-xyloside	0.606 ***	−0.018	0.686 ***	0.443 **	−0.643 ***	−0.689 ***
Phenolic acids	0.586 ***	0.084	0.687 ***	0.612 ***	−0.619 ***	−0.721 ***
Flavonols	0.594 ***	0.058	0.674 ***	0.398 **	−0.619 ***	−0.688 ***
Anthocyanins	0.634 ***	−0.042	0.690 ***	0.416 **	−0.648 ***	−0.629 ***
Total phenolics	0.612 ***	−0.004	0.697 ***	0.486 ***	−0.636 ***	−0.692 ***
**DES**	0.464 ***	0.073	0.473 ***	0.248	−0.445 **	−0.711 **
**Viscosity**	0.274	−0.191	0.207			
**Polarity**	−0.735 ***	−0.018	−0.793 ***			
**pH**	−0.616 ***	−0.027	−0.657 ***			

Correlation is significant at the 0.05 level (2-tailed); * *p* < 0.05, ** *p* < 0.01, *** *p* < 0.001

## Data Availability

The original contributions presented in this study are included in the article and Supplementary Materials. Further inquiries can be directed to the corresponding author.

## Supplementary Materials

The following supporting information can be downloaded at: https://www.mdpi.com/article/10.3390/antiox14010031/s1, Figure S1: The chromatogram of *Aronia melanocarpa* extract obtained using DESs. Peak identification: 1—neochlorogenic acid, 2—chlorogenic acid, 3—cyanidin-3-galactoside, 4—cyanidin-3-glucoside, 5*—cyanidin-3-arabinoside, 6*—cyanidin-3-xyloside, 7—quercetin-3-galactoside, 8—quercetin—3-rutinoside, 9—quercetin-3-glucoside. * tentatively identified with the help of literature data.
