# Supplementary figures and images for: Green Extraction of Phenolic Compounds from Aronia melanocarpa Using Deep Eutectic Solvents and Antioxidant Activity Investigation
Source: Antioxidants (Basel). 2024 Dec 29;14(1):31. doi: 10.3390/antiox14010031 (PMC11759870; doi:10.3390/antiox14010031)

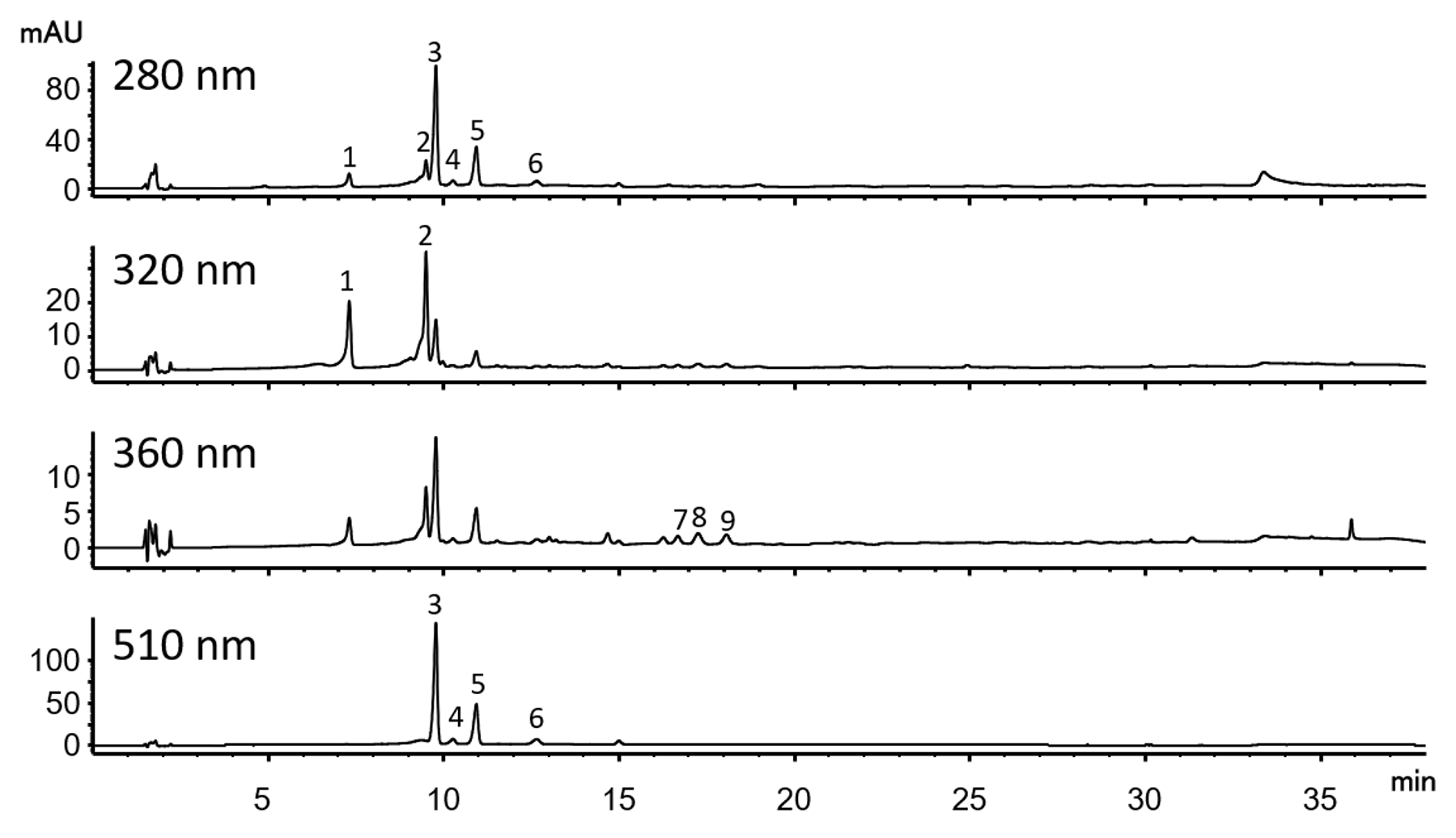

Supplement: Supplementary file 1 [file antioxidants-14-00031-s001.zip › antioxidants-3367901-supplementary.tif]
